# Using Artificial Neural Networks to Predict the Bending Behavior of Composite Sandwich Structures

**DOI:** 10.3390/polym17030337

**Published:** 2025-01-26

**Authors:** Mortda Mohammed Sahib, György Kovács

**Affiliations:** 1 Faculty of Mechanical Engineering and Informatics, University of Miskolc, 3515 Miskolc, Hungary; mortdamohammed@gmail.com; 2 Basrah Technical Institute, Southern Technical University, 61001 Basrah, Iraq

**Keywords:** composite sandwich structures, artificial neural networks, finite element model, three-point bending, experimental measurements

## Abstract

The refinement of effective data generation methods has led to a growing interest in using artificial neural networks (ANNs) to solve modeling problems related to mechanical structures. This study investigates the modeling of composite sandwich structures, i.e., structures made up of two laminated composite face sheets sandwiching a lightweight honeycomb core. An ANN was utilized to predict structural deflection and face sheet stress with low computational cost. Initially, a three-point load mode was used to determine the flexural behavior of the composite sandwich structure before subsequently analyzing the sandwich structure using the Monte Carlo sampling tool. Various combinations of face sheet materials, face sheet layer numbers, core types, core thicknesses and load magnitudes were considered as design variables in data generation. The generated data were used to train a neural network. Subsequently, the predictions of the trained ANN were compared with the outcomes of a finite element model (FEM), and the comparison was extended to real structures by conducting experimental tests. A woven carbon-fiber-reinforced polymer (WCFRP) with a Nomex honeycomb core was tested to validate the ANN predictions. The predictions from the elaborated ANN model closely matched the FEM and experimental results. Therefore, this method offers a low-computational-cost technique for designing and optimizing sandwich structures in various engineering applications.

## 1. Introduction

**Figure 1:**
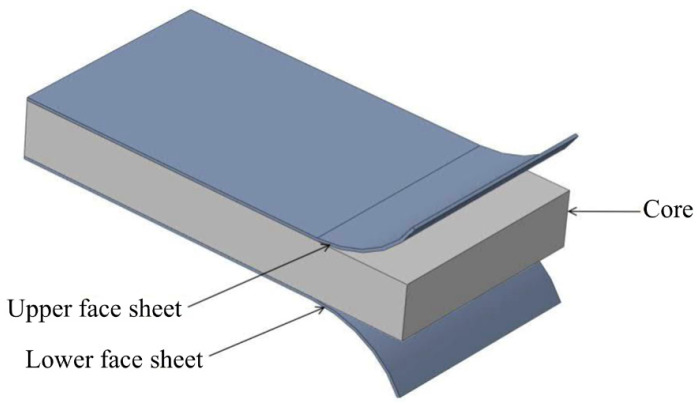
General configuration of a sandwich structure, adapted from [1].

Designed to attain minimal weight with maximum stiffness, composite sandwich structures represent a practical solution to various engineering problems. Generally, as illustrated in Figure 1, sandwich structures comprise two surface face sheets connected to a low-density core [1]. The face sheets can be classified based on the material they are made from and include fiber-reinforced plastic (FRP) laminates, fiber metal laminates (FMLs) and metals [2,3,4,5,6], while the core can be made using a foam pattern, honeycomb structure or balsa wood; it can also be functionally graded [7,8,9,10].

In regard to sandwich structure design, much research has been conducted from various perspectives. Long et al. [11] conducted a failure analysis of foam sandwich structures under impact loading using a numerical model. The model effectively captured changes in the delamination shape, and its capability to simulate the sandwich structures’ responses to impact loading was demonstrated. Wang et al. [12] conducted a theoretical study of structures with different core geometries, such as triangular, square and hexagonal honeycombs. The study aimed to improve the in-plane stiffness and yield strength of sandwich structures with various relative densities between 0.1 and 0.3. In another study by Qi et al. [13], numerical and theoretical approaches were adopted to analyze the crushing behavior of chiral honeycombs under different load types (i.e., quasi-static and dynamic loads). Hadjiloizi et al. [14] studied an asymptotic homogenization approach that can be used for the analysis of hexagonal honeycomb sandwich plates. The study suggested using unit cell models to determine the effective elastic properties of the plates.

As the outer parts of sandwich structures, the face sheets are key components that are directly influenced by external loads. Therefore, the correct selection of face sheet materials is a cornerstone of designing these structures for different engineering applications. Diverse materials are used as face sheets in sandwich structures, from isotropic metal materials to anisotropic advanced composite materials [15,16]. Given this, Zhenyu et al. [17] studied the use of titanium as a base material for both the face sheets and the core to produce a multi-disciplinary lightweight structure. The structure was fabricated using the selective laser melting printing technique. Four-point bending experiments and analytical investigations were employed to study the behavior of the structure.

Mohan et al. [18] investigated responses to low-velocity impact considering different face sheet materials, including steel, aluminum and carbon-fiber-reinforced plastic (CFRP) sheets with an aluminum foam core. The utilization of glass-fiber-reinforced plastic (GFRP) for face sheets was analyzed by Mathieson and Fam [19]. The study involved experimental and analytical investigations of the failure response. The sandwich structure was fabricated from polyurethane foam (PUF) with GFRP skins and was subjected to axial force. The bending behavior of a sandwich structure consisting of CFRP outer sheets with an aluminum honeycomb core subjected to quasi-static loading was investigated by Xiao et al. [20]. Their study concluded that the structure’s energy absorption and specific energy absorption were significantly improved when the face sheets’ fibers were oriented in the ±30° direction. Harri et al. [21] investigated using CFRP as a facing material with milled glass-fiber-reinforced rigid polyurethane foam to fabricate composite sandwich structures for aircraft applications. Amir et al. [22] examined the flexural behavior of a honeycomb sandwich structure by changing the orientation of woven glass fiber face sheets. During the investigations, the study used a three-point test with 0°, 45° and 90° fiber orientations. The authors found that the 0° fiber orientation showed superior performance in terms of maximum load capacity and flexural properties.

Recently, the potential of using fiber metal laminate (FML) as a face sheet for sandwich structures has garnered attention due to its lightweight and high-stiffness properties. Given this, Lu et al. [23] investigated the mechanical properties of a bi-material combination of metal and CFRP against loads in tensile and compressive modes. The study reported the significant influence of the structural and material parameters, overall energy absorption and yield curve. Jianxun et al. [24] investigated the dynamic behavior of aluminum honeycomb structures combined with face sheets made of glass laminate aluminum-reinforced epoxy. The study highlighted the influence of parameters like face sheet thickness, impulse loading and core stiffness on optimizing FML sandwich structures for projectile impact. A compressive strength assessment of FMLs post-impact was conducted by Patryk et al. [25]. The FML plate featured configured glass fiber/titanium and carbon fiber/titanium that had been subjected to impact energies. The study reported that delamination was the dominant damage type caused by different impact energies. The bending and impact performance of FMLs consisting of sisal-fiber-reinforced aluminum (SiRAL) laminates was analyzed by Luciano et al. [26]. The authors concluded that SiRAL holds promise as a multifunctional FML for diverse applications due to its lightweight nature and strength. Hybrid materials combining CFRP prepreg with aluminum alloy (CFRP/Al) laminates were studied by Shiyi et al. [27]. Three-point bending tests revealed that the bending properties were enhanced as the CFRP volume increased; this highlighted the significant role of CFRP in providing light and stiff laminates.

Artificial neural networks (ANNs) are inspired by how neural systems work in humans and can be used in different areas of engineering. In recent years, ANN modeling techniques have become increasingly important in various science sectors, and many ideas regarding sandwich structures and composite materials have been proposed. Lefik et al. [28,29] introduced an ANN model to predict the behavior of a homogenized composite material involving two phases of basic materials, i.e., the matrix and the fiber. The data used for training the ANN were generated from a series of analyses of an elastic–plastic model. Many research efforts have focused on using data-driven modeling techniques for sandwich structures under three-point bending. Kamarian et al. [30] applied machine learning to predict the three-point bending behavior of sandwich structures using both shallow and deep neural networks. Their study examined the sandwich beams with PLA auxetic cores and flax/epoxy face sheets reinforced with halloysite nanotubes. They demonstrated that a single-layer shallow neural network could effectively predict specific energy absorption, while a five-layer deep neural network accurately predicted load–deflection behavior. Fang et al. [31] investigated the three-point bending behavior of sandwich beams with 3D auxetic lattice cores developed through deep learning-based inverse design. The mechanical performance of the proposed sandwich structure was verified through comprehensive finite element analysis. In the field of bio-composite sandwich structures, Dashtgoli et al. [32] utilized machine learning to predict mechanical behavior under quasi-static out-of-plane loads. The experimental data were processed to train and evaluate three machine learning models: a generalized regression neural network (GRNN), extreme learning machine (ELM) and support vector regression (SVR). The GRNN demonstrated superior accuracy in capturing nonlinear load–displacement behavior compared to ELM and SVR.

Other studies have explored the use of ANNs and alternative machine learning approaches in various structural configurations and loading conditions. Yang et al. [33] used an ANN to predict the T-joint strength of sandwich structures for marine applications. The failure modes were generated numerically and the derived data processed in the ANN training model. The ANN demonstrated good agreement with the simulation results. Yong et al. [34] compared three machine learning approaches for predicting sandwich structure behavior under axial compression. The experimental data were employed to train adaptive neuro fuzzy inference system (ANFIS), artificial neural network (ANN) and simple linear regression (SLR) models. Comparative analysis revealed that the ANFIS model exhibited superior performance, followed by ANN and then SLR. Sahib and Kovacs [35] investigated a reverse design method for honeycomb sandwich structures via the use of an ANN. The Monte Carlo method was performed with governing equations to generate the training data. The reverse ANN model was more efficient and less time-consuming than traditional approaches. Akbari et al. [36] utilized a genetic algorithm with an ANN to improve the tensile strength and hardness of aluminum reinforcing particle composite sheets.

This study is organized into six sections. Section 1 is focused on the previous research efforts relevant to sandwich structures, while in Section 2 the considered materials in this study are detailed alongside their related mechanical properties. In Section 3, an elaboration on the methodologies that are used for modeling the investigated sandwich structure is provided. In Section 4, the details of the ANN and numerical and experimental results of the investigated sandwich structure are presented. In Section 5, the validation of the obtained results is introduced. In Section 6, the main conclusions from the conducted investigations are outlined.

Based on the results of our research and the reviewed literature, the main contributions of this study can be summarized as follows:

- The manufacturing and analysis of composite sandwich structures are relatively costly and complex. Therefore, the intelligent model applied in this study offers an opportunity to reduce costs and provides an accurate prediction of the flexural behavior of sandwich structures at a low computational cost, eliminating the need for tedious trial-and-error testing.
- The unique contribution of this study lies in the development of an artificial modeling system with high predictive capability that can capture the flexural behavior of various sandwich structures. For the face sheets, the structural behavior is considered in cases of hybrid face sheets combining WGFRP, WCFRP and aluminum, as well as fully FRP face sheets (e.g., WCFRP). Regarding the core, the effects of core type (e.g., Nomex and aluminum), core density and core thickness on the structural response are also considered to be captured by the elaborated ANN model.
- The validation approach expands beyond theoretical predictions and numerical simulations by using experimental tests. The validation steps for the ANN model, involving FEM analysis and experimental measurements, provide confidence that the proposed model can be used in real-world scenarios.
- This study represents a foundational advancement in the development of artificial neural network models for composite sandwich structures. In our future research, the ANN will not be limited to the flexural behavior of sandwich structures under concentrated loads and will cover other loading conditions, such as distributed and dynamic loads, thereby enhancing its adaptability for various engineering applications.

## 2. Materials of Face Sheets and Core Structural Elements

In sandwich structure design, the face sheet materials are selected based on the specific application and other factors, such as the loading conditions, lifetime requirements, availability and cost. Metal face sheets, FRP composite laminates and FMLs are all popular facing options. This study investigates common face sheet materials, offering insight into how the configuration of the sandwich structure components can influence the ultimate flexural behavior in the final structure. The materials that are considered in this work include woven carbon-fiber-reinforced plastic (WCFRP), woven glass-fiber-reinforced plastic (WGFRP) and aluminum sheets (Al).

Honeycomb cores are also widely used in sandwich structure applications. The standard geometry for these cores is a uniform hexagonal cell geometry, with the material used, cell size, wall thickness and density representing important characteristics [37]. Considering their high strength-to-weight ratio, honeycomb constructions can utilize a diverse range of materials, including aluminum, aramid paper (Nomex), plastic and fiber-reinforced plastic. Aluminum and Nomex honeycomb are the main core types for the proposed sandwich structure designs in this study.

Table 1 and Table 2 summarize the mechanical properties for the utilized materials in the investigated sandwich structures. These data serve as a foundation for the subsequent discussions and analyses presented in this work and are obtained from references [38,39,40].

## 3. Methodology

**Figure 2:**
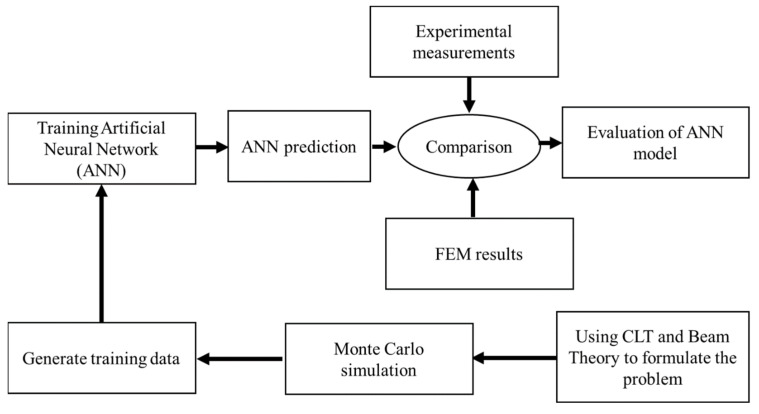
Methodology flowchart of ANN modeling in the current study.

The methodology for predicting the flexural behavior of the composite sandwich structures in the present study consists of five main steps (Figure 2):

**Step 1**: Basic structural analysis. In this step, the fundamental equations—based on beam theory and classical lamination theory—were formulated. Additionally, the range for each design variable was defined. The structural responses were set to be the maximum deflection of the sandwich structure and the maximum stress of the face sheets.

**Step 2**: Data generation. Monte Carlo sampling was utilized in this step to explore a wide range of design alternatives. The data sampling process involved executing the input space (i.e., core thickness, core density, face sheet materials and applied loads within specified ranges from Step 1) randomly and then calculating the corresponding output. This provided a comprehensive dataset capturing various design scenarios for training the ANN model.

**Step 3**: Creating and training the ANN model. The structure of the ANN model, such as the number of hidden layers, number of neurons, and training algorithm, was specified in this step. Additionally, the training process was conducted to obtain the best collaboration between the inputs and outputs.

**Step 4**: ANN performance. To check the learning efficiency of the proposed ANN, two performance metrics were used: (1) mean square error (*MSE*) and (2) coefficient of determination (*R*^2^).

**Step 5**: ANN results comparison. To assess the accuracy of the ANN, its predictions were compared with experimental results and FEM outcomes. The comparison included various sandwich structure designs in terms of the face sheet material used and core densities and thicknesses.

### 3.1. Three-Point Bending of the Investigated Structure

**Figure 3:**
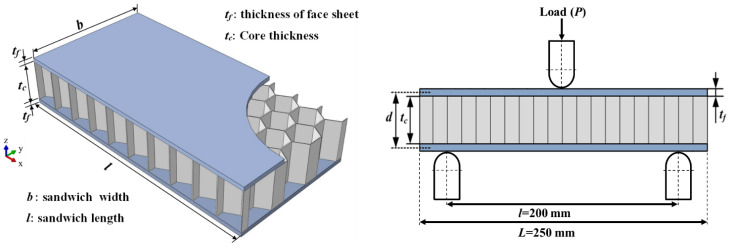
Loading configuration of the sandwich structure, adapted from [1].

To generate the required database for training the ANN model, the associated equations are solved for the sandwich structures under consideration. The three-point test is commonly employed to explore structural behavior. In this paper, sandwich structures with Al or Nomex cores and different combinations of Al-, WCFRP- and WGFRP-layered facing materials were investigated. Using a wide range of core and face sheet materials will provide insight into the structure’s behavior and diversity in terms of design alternatives. The associated loading and boundary conditions for the considered structures are presented in Figure 3, where the span length (*l*) between the supporting rollers is 200 mm, with a fixed width for the test specimen (*b*) of 50 mm.

To examine the ANN’s ability to capture the sandwich structure’s behavior, it is essential to generate a dataset with adequate diversity. Therefore, the data should cover a broad range of design alternatives for the investigated structure. The structural responses to the maximum deflection and maximum stress in the face sheets are considered in this study. Hence, the related mathematical expressions are formulated through the following equations [39,41].

#### 3.1.1. Total Deflection of the Sandwich Structure

One of the most important properties to consider in the design of composite sandwich structures is the maximum deflection. Generally, the deflection in a sandwich structure occurs in two forms: bending and shear. Of note, the out-of-plane deflection is proportional to the compressive and tensile moduli of the face sheets materials, whereas the deflection due to the shear forces is influenced by the honeycomb core’s shear modulus in the (x-z) plane. Based on beam theory calculations, the mathematical expression of the maximum deflection is as follows:

(1) $$\delta =\frac{Pl^{3}}{48D}+\frac{Pl}{4S}$$

where δ represents the total midspan deflection, *P* is the applied load and *l* is the length of the sandwich structure. The *D* and *S* terms are the bending and shear stiffnesses, respectively, which can be computed as follows:

(2) $$D=\frac{E_{f}t_{f}d^{2}b}{2}$$

(3) $$S=\frac{bd^{2}G_{xz}}{t_{c}}$$

(4) $$d=t_{f}+t_{c}$$

where *E_f_* represents the final elasticity modulus calculated by classical lamination theory (CLT) for the final laminated face sheet, *t_f_* and *t_c_* are the face sheet thickness and core thickness, respectively, *d* is the distance between the centers of the outer face sheets and *G_xz_* is the core shear modulus in the plane *xz*.

#### 3.1.2. Maximum Face Sheet Stress

Based on beam theory, the following equation can be organized to calculate the face sheet’s stress (*σ_f_*):

(5) $$\sigma_{f}=\frac{M_{max}}{dt_{f}}$$

where *M_max_* represents the maximum moment and can be determined using

(6) $$M_{max}=\frac{P\cdot l}{4}$$

### 3.2. Artificial Neural Network Modeling of the Investigated Structure

Modeling and solving of engineering problems via ANNs has become a key aspect of machine learning (ML) in different fields; however, it depends on generating data to characterize the problem. ANN models represent a sophisticated technique that can handle and address various patterns of complexity [42]. In this work, a feedforward neural network approach was employed due to its suitability for achieving broader generalization in the considered problem.

Generally, ANNs are composed of multiple simple processing elements (neurons) aligned to form layers that are interconnected via weights. The networks process input data to generate solutions, mimicking the human brain’s structure. The learning process involves adjusting parameters (weights) for each training iteration (epoch) to minimize an error function, representing the difference between the target and the ANN’s response using examples from the fed dataset. The ANN modeling in this work follows the subsequent steps.

#### 3.2.1. Data Sampling of the Sandwich Structure Under Investigation

The prediction accuracy of the ANN models is crucially influenced by the quantity of data for the training, testing and validation sets. In this analysis, the Isight 2021 software and an Excel spreadsheet are integrated. Consequently, the required data are generated by solving the governing equations of the considered sandwich structure through an Isight–Excel loop.

Monte Carlo simulation (MCS) [43] is a reliable technique that can incorporate randomness into the design process; this allows exploration of the design domain and evaluation of the sandwich structure’s responses to variations in design variables.

The design variables of the sandwich structure can be categorized as discrete or continuous. The discrete variables include core density, face sheet material and number of layers in the face sheets, where the value ranges are determined based on practical limits that cover feasible design options for the sandwich structure. The continuous variables include the core thickness and applied load, where the ranges are determined based on the design requirements of the investigated sandwich structures.

The uniform distribution type is utilized for both the discrete and continuous variables to ensure that all range values are equally likely to be processed in the Monte Carlo simulation, thus guaranteeing a comprehensive exploration of all alternatives.

In terms of the effect of the generated data on the ANN’s accuracy, the uniform distribution provides an equal representation of all possible sandwich alternatives. This, in turn, aids in the effective generalization of the ANN during the training process, improving the reliability of predictions. The design variables used in this study are illustrated in Table 3.

After running the Monte Carlo simulation, the design samples were obtained and processed as training data for the ANN. The creation of the ANN model is detailed in Section 3.2.3.

#### 3.2.2. Data Normalization Process for Training the ANN Model

In ANN applications, data normalization is a key part of achieving balanced data representation and ensuring training convergence. During this process, the input data are converted into a scaled range. This helps avoid the dominance of certain features during the ANN learning process. Consequently, data that are properly scaled enable efficient ANN training. In this paper, the normalization process scales the generated data to the range [0.1, 0.9] by applying the below equation [44]:

(7) $$x_{i}=\lambda_{1}+\left(\lambda_{2}-\lambda_{1}\right)\left(\frac{z_{i}-z_{min}}{z_{max}-z_{min}}\right)$$

where $\lambda_{1}$ and $\lambda_{2}$ are the upper and lower values for the normalized parameters, respectively, *x_i_* represents the scaled value for the specific parameter and *z_i_* is the original value of the parameter. Additionally, *z_min_* and *z_max_* are the minimum and maximum scaled values, respectively.

#### 3.2.3. Creating ANN Model for the Investigated Sandwich Structure

The focus of this study is on the loading conditions rather than the failure limits. Therefore, the analytical models adopted are based on the sandwich structure behavior within elastic limits. Consequently, only combinations of loads that induced maximum deflection in the structure below the failure threshold were taken into account.

**Figure 4:**
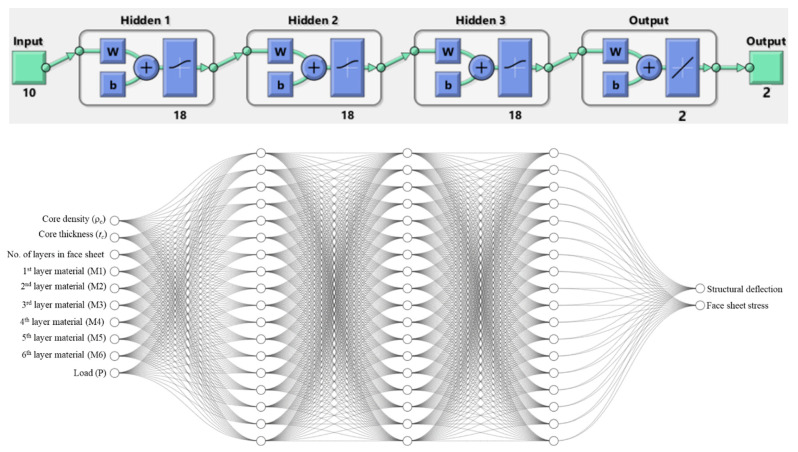
Structure of the neural network for the structure under investigation.

Thus, the extracted sampling data from Monte Carlo simulation consisted of 6000 sandwich structure design points, with associated structure responses in terms of structure deflection and maximum face sheet stress. In general, the architecture of a backpropagation feedforward ANN comprises an input layer for receiving inputs, one or more hidden layers for executing the training process and an output layer for producing predictions. Each layer is fully interconnected with the next layer. In this study, as illustrated in Figure 4, the input layers consisted of 10 neurons representing the input variables (i.e., core density, core thickness, face sheets materials and applied load), whereas three hidden layers were used with 18 neurons each, and the output layer included 2 neurons for the maximum deflection and maximum face sheet stress.

The Bayesian regularization (BR) backpropagation algorithm was employed to train the ANN model. Generally, the BR algorithm is not included in the validation set, as it has a built-in validation function to determine the optimal parameters during the training process [45]. Therefore, the data were divided randomly into two sets, with 60% allocated to train the model and 40% reserved for testing. Figure 4 illustrates the ANN architecture of the sandwich structure investigation.

**Figure 5:**
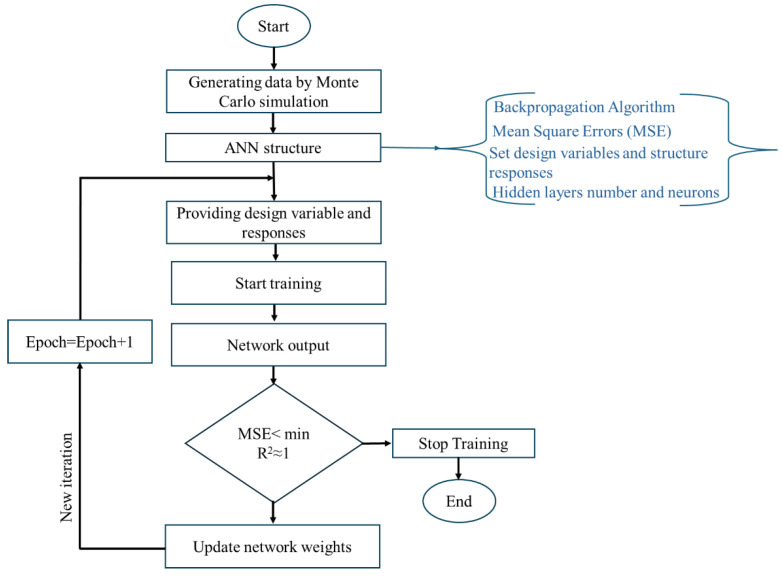
Flowchart of the fundamental steps required to build the ANN model.

The accuracy of the ANN model’s prediction was evaluated using the mean square error (*MSE*) (Equation (8)). Additionally, the determination coefficient (*R*^2^) was calculated to quantify the accuracy fitness between the ANN model prediction and the actual data (Equation (9)) [46]. Models with a higher *R*^2^ (typically *R*^2^ > 0.98~0.99) have greater predictive capability, while those with lower MSE values have greater accuracy.

(8) $$MSE=\frac{1}{n}\sum_{i=1}^{n}{\left(x_{act,i}-x_{pred,i}\right)}^{2}$$

(9) $$R^{2}=1-\frac{\sum_{i=1}^{n}{\left(x_{act,i}-x_{pred,i}\right)}^{2}}{\sum_{i=1}^{n}{\left(x_{act,i}-x_{avg}\right)}^{2}}$$

where *n* denotes the number of data points, $x_{act,i}$ represents the actual data point, $x_{pred,i}$ represents the predicted value obtained from the established network and $x_{avg}$ denotes the mean of the $x_{act,i}$ values. Figure 5 illustrates the flowchart depicting the fundamental steps to create the neural network model.

### 3.3. Numerical Modeling of the Investigated Sandwich Structure

Due to the difficulty of modeling all the design alternatives via FEM, some of the investigated designs were simulated numerically to compare the FEM with the ANN prediction and test measurements related to the flexural behavior of the composite sandwich structure. The ABAQUS CAE 2017 [47] software was used for modeling the three-point bending configuration. The material properties were as described in Section 2.

Initially, the modeling used a hybrid face sheet with six layers: two layers of WCFRP, two layers of WGFRP and two layers of aluminum. Additionally, to mimic the experimental test, four additional structural configurations were modeled, which included 3–6 layers of WCFRP in the face sheets combined with a Nomex honeycomb core. The dimensions were 250 mm × 50 mm. The face sheets were modeled as continuum shell elements (S4R), and, from the composite module, a number of layers was assigned to the face sheets. Meanwhile, to improve the computational efficiency of the FEM model, the honeycomb core was approximated as a homogeneous solid layer with consistent mechanical properties [48].

**Figure 6:**
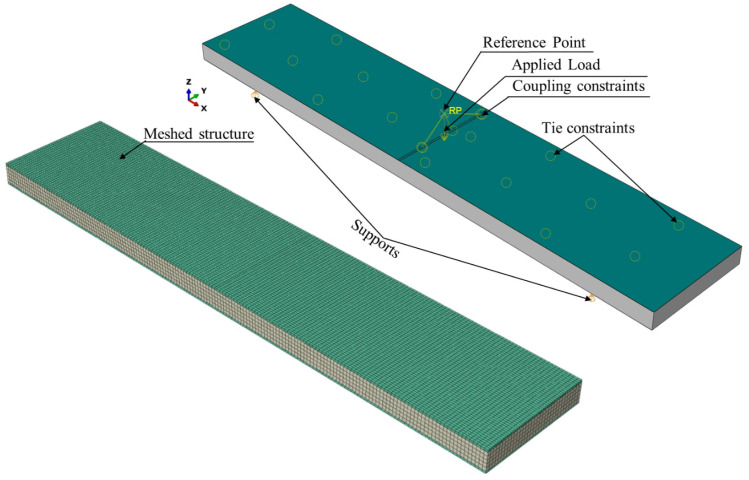
FEM modeling for the sandwich structure being investigated.

The investigated structure’s supports were modeled to be simple supports, and a reference point (RP) was created for applying the load to the upper face sheet. A tie interaction was specified for the mating surfaces (i.e., face sheets and core), while a kinematic coupling constraint was defined between the reference point and the loading region in the upper face sheet. This constraint couples the motion of the reference point to the motion of the corresponding regions on the structure, which in turn allows the applied load to be effectively transferred from the reference points to the modeled structure. The interaction, loading and boundary conditions are depicted on the right-hand side of Figure 6. To solve the numerical model, the meshing process was conducted with 27,555 elements for the core. The shell mesh was assigned for the face sheets with approximately 8500 elements, as depicted in the meshed structure shown in Figure 6. It is noteworthy that the mid-plane deflection was evaluated as a function of the applied load. Therefore, only the stresses within the elastic portion were considered in these analyses.

### 3.4. Experimental Setup of the Investigated Sandwich Structure

We focused on investigating sandwich structures under out-of-plane loading conditions. This is due to the extensive range of engineering parts, such as components of trains, airplanes and vehicles, that experience this loading mode. Stiffness, weight and cost need to be considered when designing these parts. Given this, one goal of this study was to provide insight into sandwich structures from an experimental perspective.

**Figure 7:**
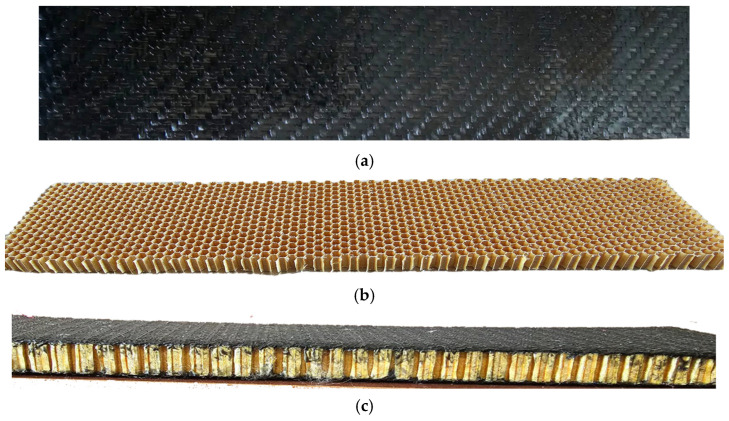
The investigated sandwich structure’s components: (**a**) face sheet, (**b**) honeycomb core and (**c**) assembled final structure.

The sandwich structure consisting of WCFRP-laminated face sheets combined with a Nomex honeycomb core was investigated (Figure 7). To provide the required strength in the core–face sheet connection regions, an epoxy adhesive was applied between the core and face sheets.

#### 3.4.1. Manufacturing the Investigated Test Specimens via the Vacuum Bag Technique

**Figure 8:**
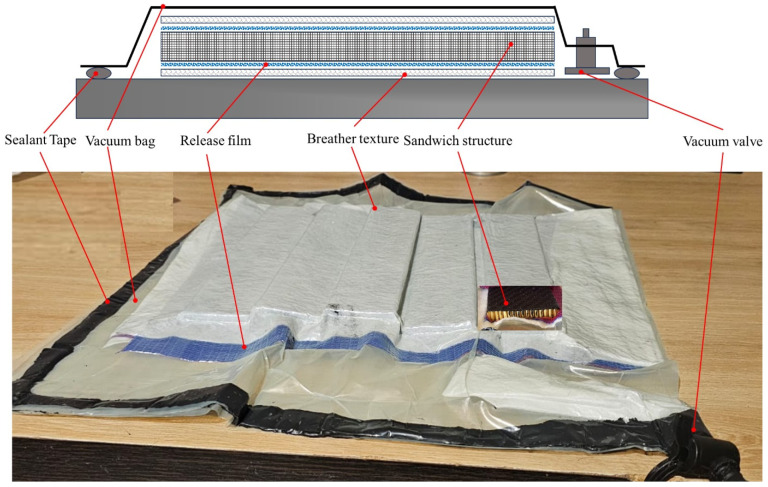
Vacuum bag applied during the manufacturing of the test specimens.

The vacuum bag technique is a commonly used approach for creating laminated composite structures. The vacuum bag components include the release film, breather, nylon bag sealant tape and vacuum valve, as illustrated in Figure 8. The release film covers both sides of composite structure to prevent it from sticking to the breather or the mold. The breather (or bleeder) texture helps to distribute the vacuum and absorb excess resin during the autoclaving process. The final part is flexible nylon sealing tape, which is used to form tight seals that prevent leakage and allow the required vacuum to be obtained.

The sandwich structure components (i.e., laminated prepreg face sheets, core and adhesive) were sliced into the standard dimensions (length of 250 mm and width of 50 mm) according to ASTM C393/C 393M [49]. The laminated face sheets were made from layering layers of carbon fiber prepreg and then attaching them to the Nomex honeycomb core. Afterwards, the assembled parts were placed in the vacuum bag.

By applying vacuum inside the bag through a vacuum pump, a uniform pressure is induced over the assembled sandwich structure. This helps to remove trapped gases, improve the stacking of the face sheet layers and enhance the adhesion of the face sheets to the core. After confirming there were no leaks in the bag, the assembled sandwich structure was cured in an autoclave.

**Figure 9:**
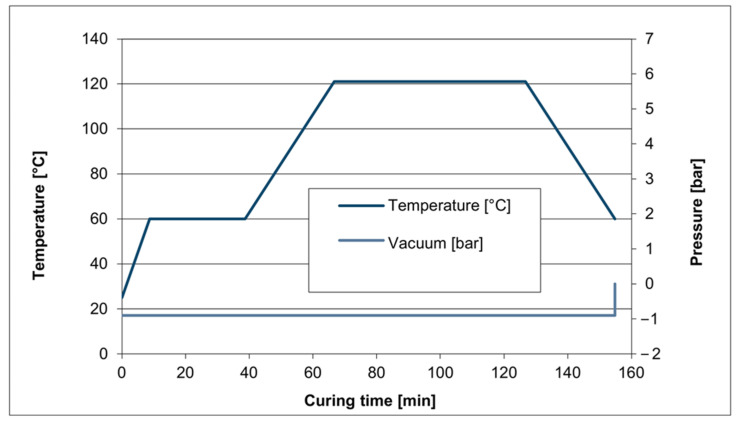
Curing cycle for the prepreg of the face sheets (manufacturer’s protocol).

To achieve this, the curing time was 150 min, and, according to the manufacturer’s protocol, the highest temperature reached was 123 °C. The implemented curing profile for the prepreg in this study is illustrated in Figure 9. The resin viscosity rapidly decreases as the temperature increases; this indicates the initiation of a chemical reaction within the resin.

After approximately 70 min of pre-heating, the main curing phase begins, which includes holding the temperature at 123 °C for 60 min. At this point, the resin’s viscosity reaches a minimum as it transforms to a solid phase. Importantly, the vacuum is applied through all curing stages to provide a uniform pressure on the composite structure and remove any generated volatiles. After completing the main curing step, the autoclave is switched off to enable a gradual cooling.

#### 3.4.2. Experimental Work Configuration

The manufactured test specimens, produced via vacuum bag technology as described in Section 3.4.1, were used in a three-point bending test. The goal of the test was to investigate the flexural behavior of the designed sandwich structure and compare the results with ANN predictions. This was assessed by analyzing the established load–displacement curves. Based on the number of layers in the face sheets, four groups of sandwich structure test specimens were manufactured, those with (1) three layers, (2) four layers, (3) five layers and (4) six layers. Each group included three test specimens. A Nomex honeycomb core with a density of 48 kg/m^3^ and an 8 mm thickness was used as the core material for all specimens. The length of specimens was specified to be a working span of 200 mm plus 50 mm based on the ASTM standard [44,49], while the width of the specimens was fixed at 50 mm.

**Figure 10:**
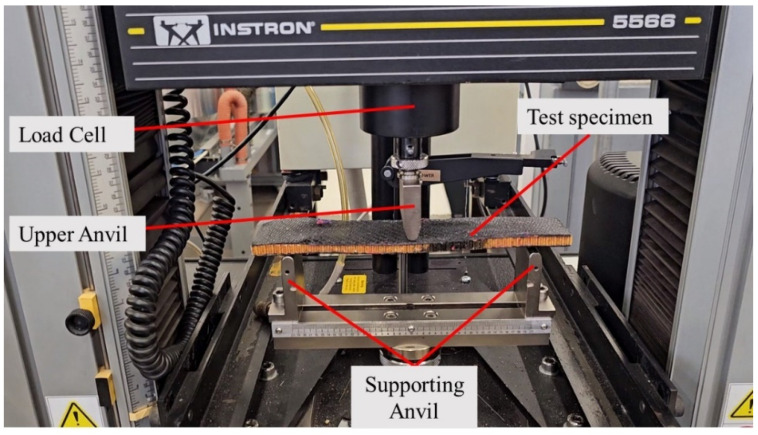
The three-point test setup for obtaining the force-displacement of the sandwich test specimens.

The three-point bending test was carried out using an Instron 5566 (Instron, Canton, MA, USA) universal testing machine, as shown in Figure 10. During the test, the machine crosshead was fixed at a rate of 3 mm/min. Each sandwich specimen was loaded until the maximum load that it could sustain was reached. During the test, the load data and the derived deflections were recorded by the machine’s data acquisition system.

**Figure 11:**
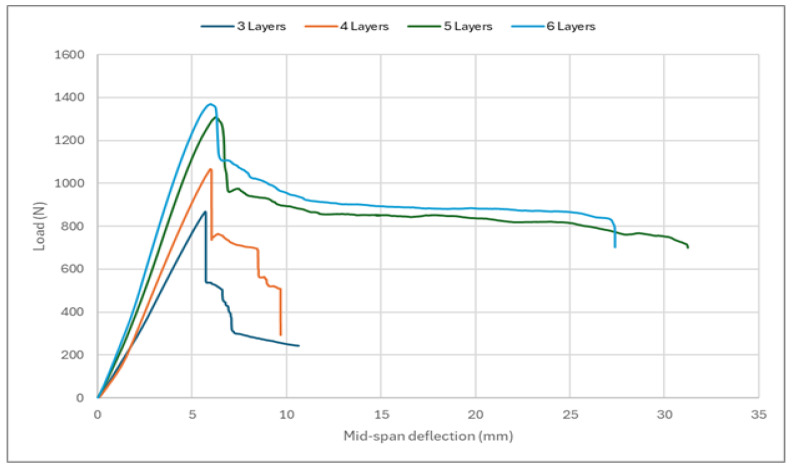
Force–displacement curves for the tested sandwich structures.

Figure 11 illustrates the load–displacement curves obtained for the respective test specimens. These curves provide an in-depth understanding of the flexural behavior of the investigated sandwich structures. By analyzing these curves, the effect on the overall structural performance of changing the number of layers assigned to the face sheets can be evaluated.

Generally, the specimens exhibited similar behavior, with the curves initially displaying a linear increase in load and displacement. However, after a certain load value was reached, the structure went through a rapid deterioration due to face sheet and core failures. As shown in Figure 11, adding more composite layers to the face sheets results in a stiffer structure.

## 4. Results of ANN and FEM Analysis on Investigated Sandwich Structures

In this section, the ANN model’s performance is assessed in terms of *MSE* and *R*^2^. To further assess the ANN’s ability to capture the behavior of complex sandwich structure designs, face sheets with FML (hybrid)-laminated configurations combined with Nomex and Al honeycomb cores in different thicknesses are modeled using FEM. Finally, the FEM results are compared with the ANN predictions for the same sandwich structure designs. The details of these analyses will be further explained in the following subsections.

### 4.1. Artificial Neural Network Performance of the Investigated Sandwich Structure

**Figure 12:**
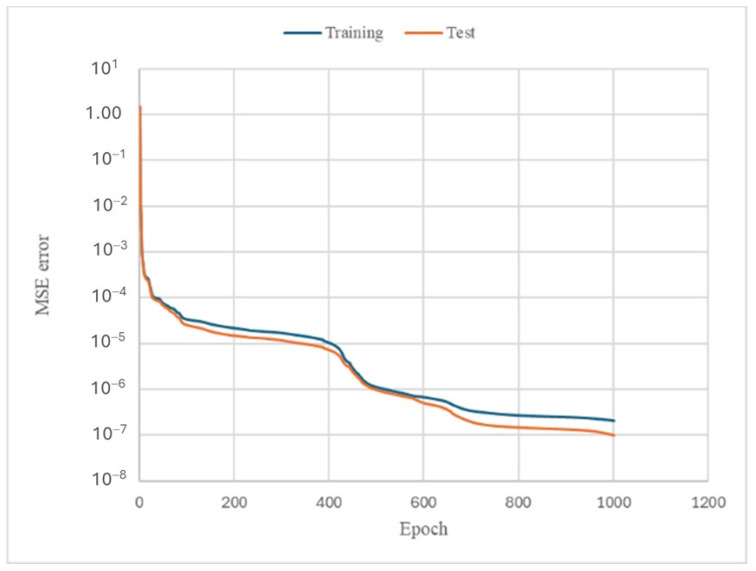
Mean square error (*MSE*) values for the training artificial neural network.

The ANN model’s performance was evaluated via the MSE. Additionally, *R*^2^ was calculated to check the similarity between the ANN predictions and the experimental data. Initially, both the training and testing errors began with high values, indicating a big difference between the ANN prediction and the actual values. With further training iterations (epochs), the *MSE* underwent a rapid decrease. This indicated that the ANN was learning effectively in order to provide a better fit with the training data. The final ANN model exhibited a very low *MSE* value of about 1 × 10^−7^ after 1000 epochs, as can be seen in Figure 12.

**Figure 13:**
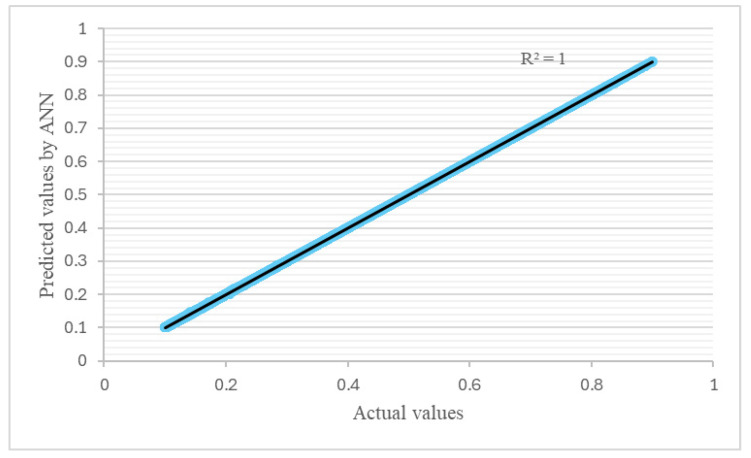
Artificial neural network predictions with actual values (training).

**Figure 14:**
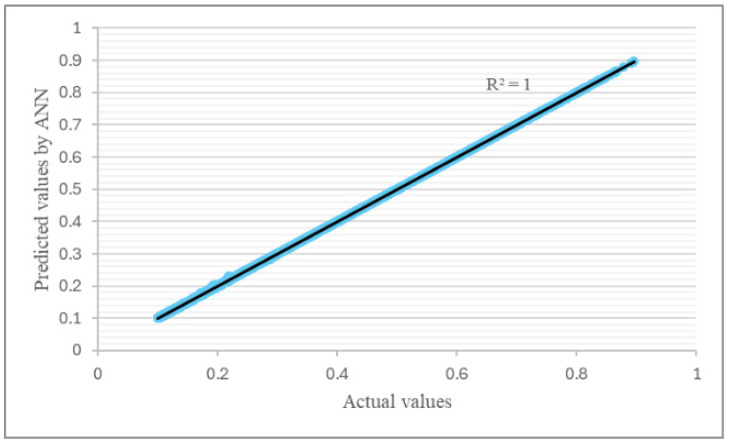
Artificial neural network predictions with actual values (test).

The linear relationship depicted for the determination coefficient (*R*^2^) indicates a strong predictive ability and signifies better accuracy. Figure 13 and Figure 14 depict the training and test set correlations between the values predicted via the ANN and the target values used for creating the ANN model. Based on these performance results, it can be concluded that the trained ANN acquired the ability to effectively model the investigated sandwich structures.

### 4.2. Finite Element Model for the Sandwich Structure with FML Face Sheets

Due to the difficulty of modeling all alternative designs numerically, FEM analysis was conducted for some complex structural designs. In this section, FEM modeling was used for a sandwich structure with face sheets consisting of six layers, as this configuration experienced the maximum applied load. The FML (hybrid)-laminated face sheets are considered as having the selected materials equally divided within them (i.e., two layers of WCFRP, two layers of WGFRP and two layers of Al). The cores are either Nomex or Al honeycomb. The FEM modeling was performed to obtain the structural deflection and stress distribution across the face sheets in the three different sandwich structures. Details of the three sandwich structures can be found in Table 4 and are described as follows:

Design 1: FML hybrid face sheets (2WCFRP + 2WGFRP + 2Al layers) with Nomex honeycomb core (*t_c_* = 8 mm);

Design 2: FML hybrid face sheets (2WCFRP + 2WGFRP + 2Al layers) with Nomex honeycomb core (*t_c_* = 12 mm);

Design 3: FML hybrid face sheets (2WCFRP + 2WGFRP + 2Al layers) with Al honeycomb core (*t_c_* = 8 mm).

**Figure 15:**
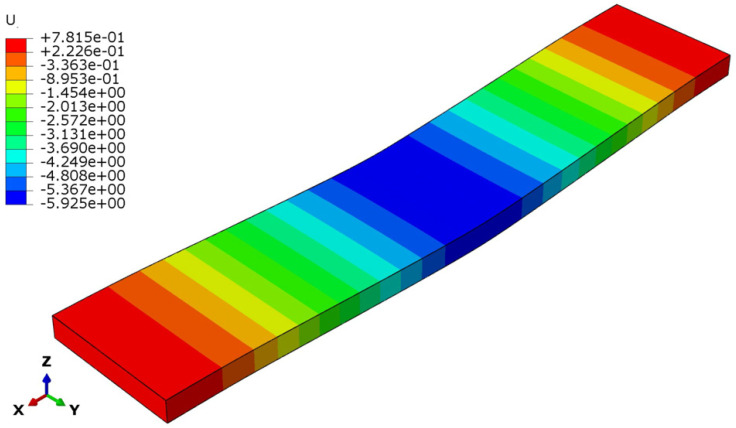
Structural deflection in Design 1.

**Figure 16:**
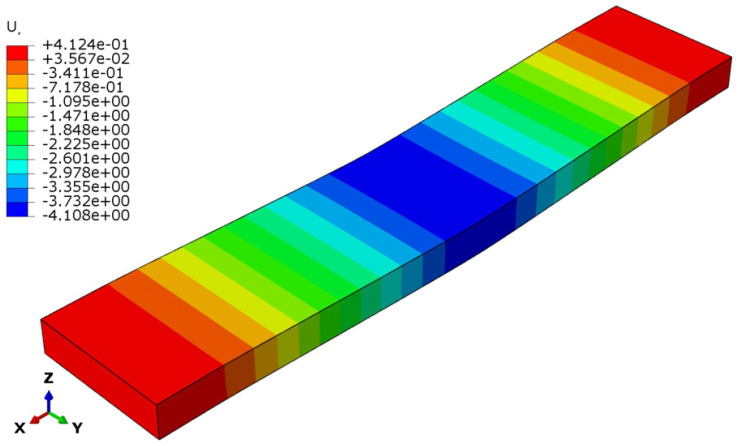
Structural deflection in Design 2.

**Figure 17:**
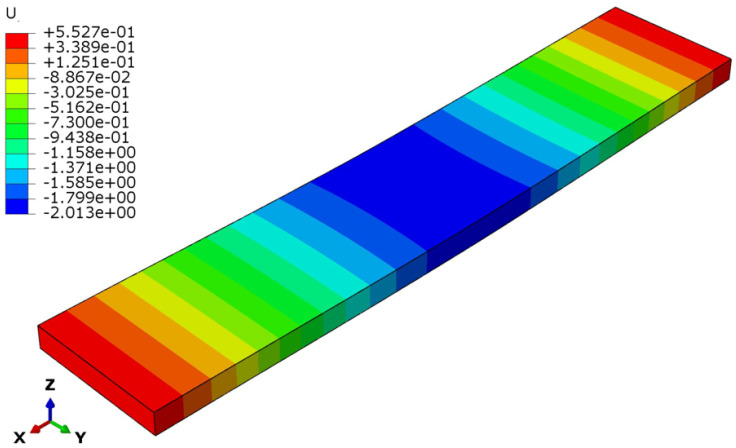
Structural deflection in Design 3.

**Figure 18:**
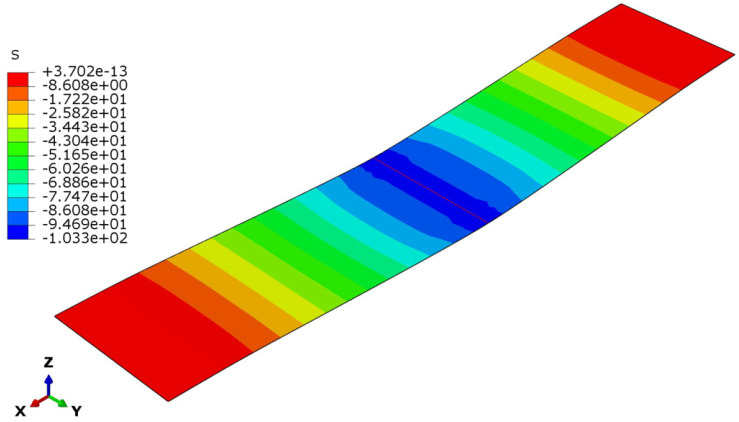
Face sheet stresses in Design 1.

**Figure 19:**
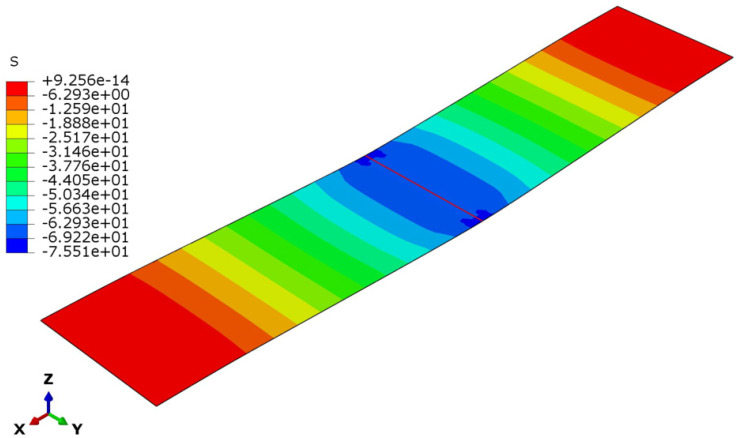
Face sheet stresses in Design 2.

**Figure 20:**
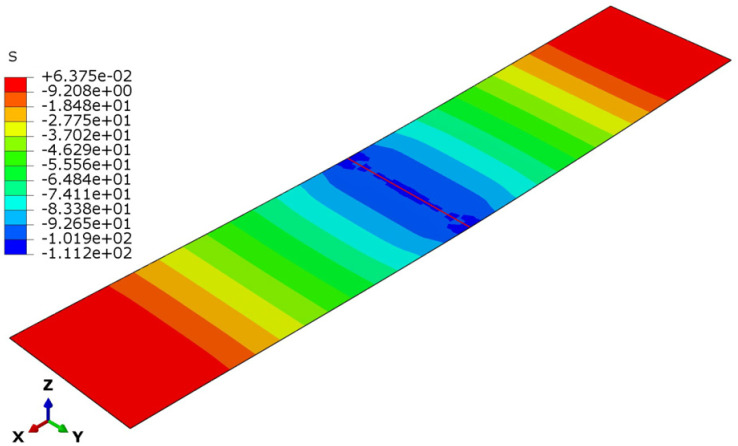
Face sheet stresses in Design 3.

The deflection and stress patterns in Figure 15, Figure 16, Figure 17, Figure 18, Figure 19 and Figure 20 provide insight into the structural behavior of the investigated structural alternatives.

From Figure 15, Figure 16 and Figure 17, Designs 1–3 exhibit a similar deflection mode, which is characterized by global bending; the maximum deflection occurs at the mid-span of the structure. As can be observed in Figure 15, the deformation mode indicates that Design 1—which includes a honeycomb core with a lower thickness and density (*t_c_* = 8 mm, ρ_c_ = 48 kg/m^3^)—resulted in higher structural deflection. This behavior reflects a lower flexural modulus from a structural performance perspective. Design 2, the structure with a thicker honeycomb core (*t_c_* = 12 mm, ρ_c_ = 48 kg/m^3^), exhibited lower deflection, as shown in Figure 16. This can be attributed to the fact that flexural stiffness is directly proportional to core thickness. Figure 17 illustrates the deflection mode for Design 3 (*t_c_* = 8 mm, ρ_c_ = 83 kg/m^3^). It demonstrates that a higher core density increases the structural stiffness, resulting in reduced overall deflection.

The face sheets’ stress modes for the three designs are illustrated in Figure 18, Figure 19 and Figure 20. For Design 1, with a thinner core with lower density, the core’s contribution to stiffness and load distribution is minimal. Given this, the bending moment concentrated more stress on the face sheets (see Figure 18), as they bear a significant portion of the load. Increasing the core thickness in Design 2 improved the sandwich structure’s flexural stiffness. The thicker core shifts more of the load away from the face sheets and distributes it across a larger cross-sectional area of the structure. This reduces the bending stresses in the face sheets (see Figure 19), despite the core density remaining the same. Lastly, for Design 3 (with the denser core), the core becomes stiffer and more resistant to deformation. However, the thickness is insufficient to achieve load redistribution in an efficient manner. A denser core transfers higher reaction forces between the applied load and the face sheets, causing the face sheets to experience increased stresses (see Figure 20).

### 4.3. Comparison of FEM Results and ANN Predictions in FML Face Sheet Sandwich Structures

A comparison between the FEM results and the ANN model’s predictions is presented in Table 5.

The ANN predictions and FEM results are very similar, with a maximum difference of 8.1%. This alignment demonstrates the remarkable accuracy of the ANN model’s predictions and indicates that the ANN model effectively captured the behavior exhibited by the composite sandwich structures across various design configuration complexities.

It is worth noting that the similarity between the FEM results and ANN predictions, as well as the observed behavior patterns, prove the reliability of the ANN model in predicting the performance of different composite sandwich structures.

## 5. Validation of the Elaborated ANN Model with Experimental Measurements and FEM Results

Due to limitations in manufacturing the FML face sheets and assessing the ANN model using a single face sheet material with different numbers of layers, the test specimens that were used for the validation were made from laminated WCFRP face sheets with a varying number of layers (3–6 layers in the laminate), while the core was Nomex (48 kg/m^3^) with a thickness of 8 mm.

Additionally, the same experimental test specimens (see Figure 6) were modeled numerically in terms of dimensions, materials, boundary conditions and loading conditions. Consequently, the maximum sandwich deflection and maximum face sheet stresses were extracted from the FEM results.

**Figure 21:**
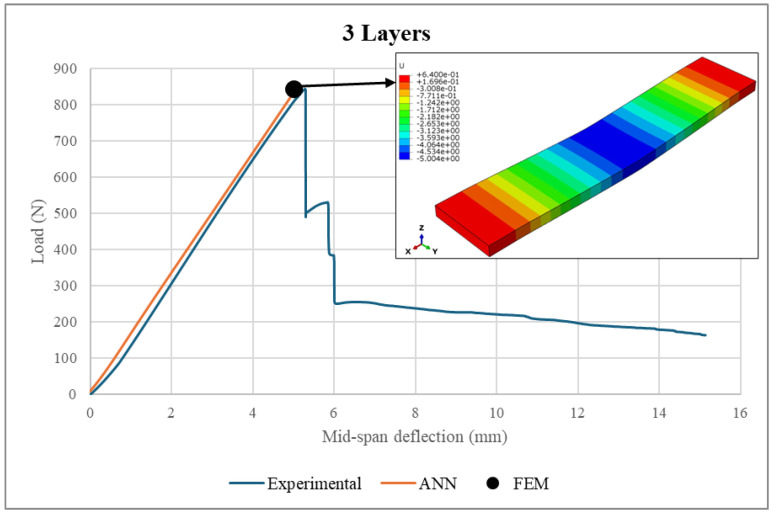
Load–deflection curve in a 3-layer face sheet structure.

**Figure 22:**
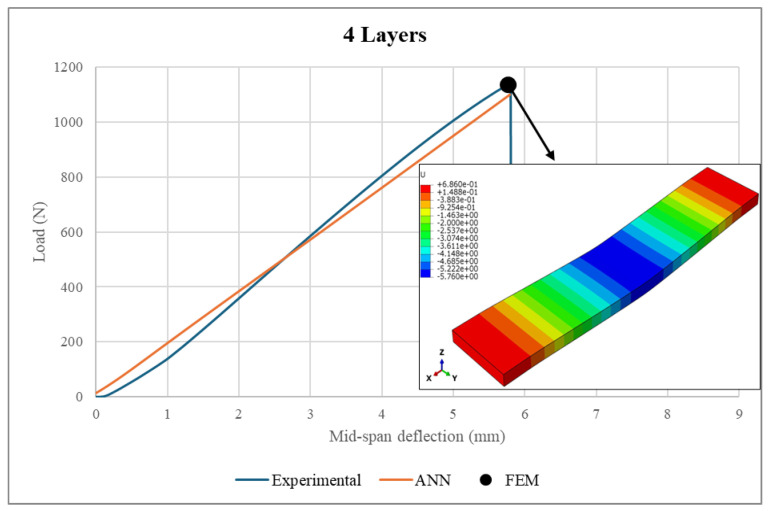
Load–deflection curve in a 4-layer face sheet structure.

**Figure 23:**
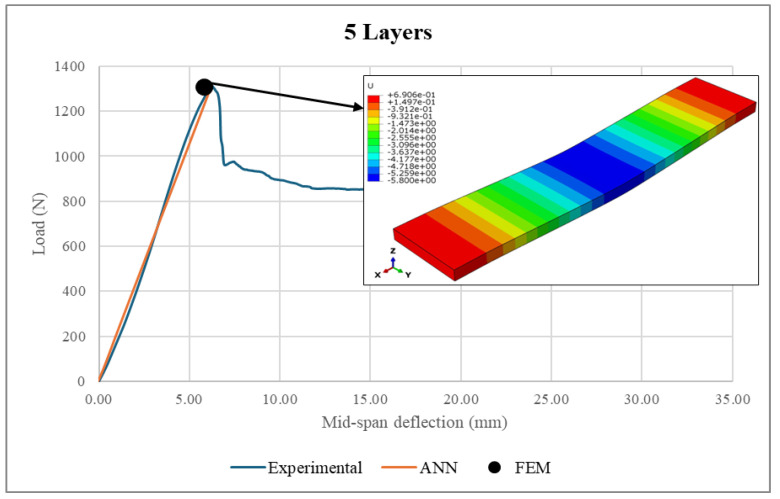
Load–deflection curve in a 5-layer face sheet structure.

**Figure 24:**
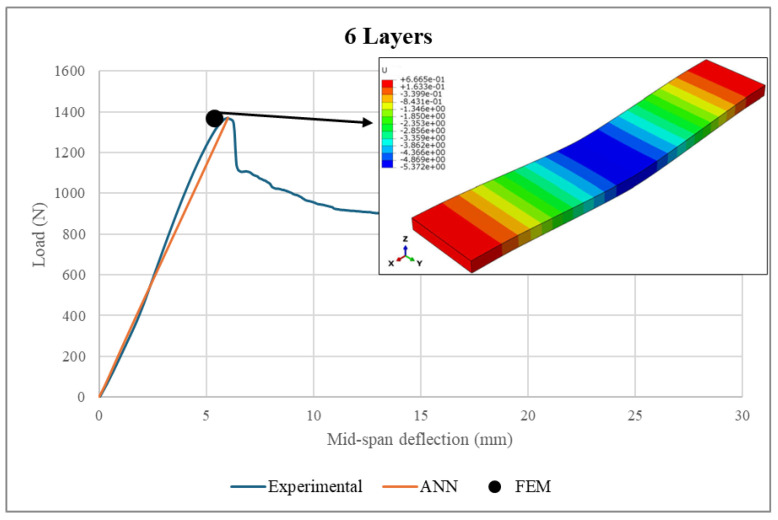
Load–deflection curve in a 6-layer face sheet structure.

A comprehensive validation of the ANN model against experimental measurements and FEM analysis was performed. Firstly, the experimental loads were fed into the ANN model to predict the load–deflection curves. Then, the ANN predictions were compared to the corresponding experimental measurements and FEM results, as illustrated in Figure 21, Figure 22, Figure 23 and Figure 24. It is worth noting that the experimental data used in FEM modeling pertained to the elastic stage of the structural behavior, i.e., before the structural failure threshold.

Based on Figure 21, Figure 22, Figure 23 and Figure 24, there is excellent agreement between the ANN predictions and the experimental measurements and FEM results.

For clarity and better understanding, Table 6 summarizes the ANN predictions at the maximum load point, including the associated differences between the ANN predictions, real experimental measurements and FEM results for the sandwich structure deflection and face sheet stress.

As can be seen from the table, the deflection values exhibited similar patterns, where the sandwich structures with face sheets comprising 3–6 layers were associated with comparable deflections. However, it is important to mention that the maximum applied loads were directly proportional to the number of layers in the face sheets. For example, the maximum sustained load was increased from 842.7 N to 1370.1 N as the number of face sheet layers increased from 3 to 6. In conclusion, the makeup of the face sheets has a significant influence on the total structural deflection. Meanwhile, the maximum stresses achieved by the face sheets were proportional to the number of layers within them. An increase in the number of WCFRP layers resulted in a reduction of stress in the face sheets. This can be explained by the enhancement of the load-carrying capacity, which was achieved by adding more layers to the face sheets. Therefore, thicker face sheets not only improve the stiffness of the structure but also provide lower stress concentrations and hence minimize the risk of structural failure under applied loads.

The ANN predictions in terms of maximum structural deflection and maximum face sheet stresses were also compared with the experimental measurements and FEM results. For structural deflection, the maximum deviations were approximately 4.16% and 10.5%, respectively. For the face sheets stresses, the ANN predictions showed good alignment with the FEM results, as the maximum difference was 6.21%. This behavior was consistent across all of the considered numbers of layers, underscoring the robustness of the ANN model.

## 6. Conclusions

This study investigated the application of an ANN for modeling composite sandwich structures. The analysis began by solving the analytical equations used during the design of the investigated structures under three-point bending. The design formulation was processed via MCS to generate a training dataset. Consequently, an ANN model was developed to address the composite sandwich structure problem under investigation. The data generated included different configurations of laminated face sheets, face sheet materials, honeycomb core types and applied loads. Subsequently, an artificial neural network was trained with the data from the previous step. A network model with three hidden layers, a Bayesian regularization (BR) backpropagation algorithm and 18 neurons for each hidden layer showed good prediction performance. The developed ANN model exhibited good accuracy in predicting the flexural behavior of the investigated sandwich structure. An FEM analysis was performed for the investigated sandwich structure with FML (hybrid) face sheets, and experimental measurements for the sandwich structure under three-point bending were conducted. The developed ANN demonstrated good agreement with FEM results and experimental measurements.

Consequently, the proposed ANN model has proved to be a reliable tool for structural engineering applications, offering an effective alternative to traditional modeling methods. This highlights the main added value of this study, which is the development of a detailed framework for utilizing ANNs in sandwich structure analysis, data generation, model training and validation procedures. The current study bridges the gap between theory and practice, as it integrates computational models (ANN and FEM) with experimental validation, creating a valuable link between theoretical predictions and real-world structural behavior.

In conclusion, this study provides a framework that can be used as a reference for further research efforts and promotes the integration of machine learning techniques into structural engineering.

## Figures and Tables

**Table 1:** Engineering properties of face sheets applied in the sandwich structures.

Material Properties	Values		
	Al	WGFRP	WCFRP
Longitudinal modulus: *E_x_* [MPa]	70,000	20,000	70,000
Transverse modulus: *E_y_* [MPa]	70,000	17,000	60,000
In-plane shear modulus: *G_xy_* [MPa]	26,000	3500	4500
Major Poisson’s ratio: *ν_xy_* [-]	0.33	0.13	0.05
Density: *ρ_f_* [kg/m^3^]	2780	1.88	1.5
Lamina thickness: *t_l_* [mm]	0.2	0.25	0.23
Longitudinal tensile strength: *σ_xt_* [MPa]	186	600	800
Longitudinal compressive strength: *σ_xc_* [MPa]	186	600	800
Transverse tensile strength: *σ_yt_* [MPa]	186	550	700
Transverse compressive strength: *σ_yc_* [MPa]	186	550	700
In-plane shear strength: *σ_xy_* [MPa]	110	55	60

**Table 2:** Honeycomb core properties.

Density	Mechanical Properties in *x* Direction		Mechanical Properties in *y* Direction		Mechanical Properties in *z* Direction	
*ρ_c_* [kg/m^3^]	Strength: *σ_xz_* [MPa]	Modulus: *G_xz_* [MPa]	Strength: *σ_yz_* [MPa]	Modulus: *G_yz_* [MPa]	Strength: *σ_zz_* [MPa]	Modulus: *E_zz_* [MPa]
Al honeycomb						
29	0.4	55	0.65	110	0.9	165
37	0.45	90	0.8	190	1.4	240
42	0.5	100	0.9	220	1.5	275
54	0.85	130	1.4	260	2.5	540
59	0.9	140	1.45	280	2.6	630
83	1.5	220	2.4	440	4.6	1000
Nomex honeycomb						
29	0.28	12	0.52	22	0.54	17
48	0.62	24	1.16	38	1.9	25
64	0.82	30	1.48	50	3.7	35
80	1.05	38	1.95	68	4.7	40
96	1.42	56	2.45	86	6.6	50
123	1.76	71	2.9	98	10	60

**Table 3:** Design variables of the investigated sandwich structure.

Design Variables		Remarks
Core density	$\rho_{c}[kg/m^{3}]$	Al and Nomex cores in Table 2
Core thickness	$5\leq t_{c}\leq 18$ [mm]	Continuous value
Face sheet materials	WCFRP layer: specified as No. 1 WGFRP layer: specified as No. 2 Aluminum layer: specified as No. 3	Integer values, discrete variable
Layer number	*N_l_*: 3, 4, 5 or 6 [layers]	Integer values, discrete variable
Applied load	100≤P≤2000[N]	Continuous value

**Table 4:** Design configurations for sandwich structures with FML face sheets.

Structural Alternatives (Design No.)	Face Sheet Composition FML (Hybrid)	Core Type (*ρ*) [kg/m^3^]	Core Thickness (*t_c_*) [mm]	Load (*P*) [N]
Design 1	2 layers WCFRP + 2 layers WGFRP + 2 layers Al (same face sheets in each of the 3 alternatives)	Nomex honeycomb core 48	8	1370.4
Design 2		Nomex honeycomb core 48	12	
Design 3		Al honeycomb core 83	8	

**Table 5:** Comparison between ANN predictions and FEM results.

Structural Alternatives (Design No.)	Maximum Deflection [mm]			Maximum Stress in the Face Sheet [MPa]		
	ANN Prediction	FEM	Difference [%]	ANN Prediction	FEM	Difference [%]
**Design 1** Face sheets: FML (Hybrid) Core type: Nomex (*ρ* = 48 kg/m^3^) Core thickness (*t_c_*): 8 mm	6.45	5.925	8.1	107.4	103.3	3.8
**Design 2** Face sheets: FML (Hybrid) Core type: Nomex (*ρ* = 48 kg/m^3^) Core thickness (*t_c_*): 12 mm	4.42	4.1	7.2	75.23	75.5	0.4
**Design 3** Face sheets: FML (Hybrid) Core type: Al (*ρ* = 83 kg/m^3^) Core thickness (*t_c_*): 8 mm	2.09	2.01	3.8	107.44	111.2	3.5

**Table 6:** Comparison of experimental measurements with ANN model for the investigated structure.

No. of Layers in the Face Sheets	Applied Load [N]	Maximum Deflection					Face Sheet Stress		
		Experimental [mm]	FEM [mm]	ANN [mm]	Error (ANN vs. Exp. Test) [%]	Error (ANN vs. FEM) [%]	FEM [MPa]	ANN [MPa]	Error (ANN vs. FEM) [%]
**3**	842.691	5.28	5.00	5.064	4.16	1.26	150.1	140.7	6.21
**4**	1137.008	5.78	5.76	5.983	3.51	3.73	145.0	138.7	4.3
**5**	1310.127	6.29	5.80	6.199	1.54	6.44	122.6	124.1	1.26
**6**	1370.138	6.00	5.37	6.006	0.11	10.59	107.6	106.0	1.42

## Data Availability

Data are contained within the article.
